# Tetanus Toxin C-Fragment: The Courier and the Cure?

**DOI:** 10.3390/toxins2112622

**Published:** 2010-10-29

**Authors:** Janne M. Toivonen, Sara Oliván, Rosario Osta

**Affiliations:** LAGENBIO-I3A, Veterinary School, Aragón Institute of Health Sciences (IACS), Universidad de Zaragoza, Miguel Servet 177, 50013 Zaragoza, Spain; Email: jtoivonen.iacs@aragon.es (J.M.T.); soligar@unizar.es (S.O.)

**Keywords:** tetanus toxin C-fragment, therapeutic molecules, gene therapy, retrograde transport, neurodegenerative disease, motor neuron disease

## Abstract

In many neurological disorders strategies for a specific delivery of a biological activity from the periphery to the central nervous system (CNS) remains a considerable challenge for successful therapy. Reporter assays have established that the non-toxic C-fragment of tetanus toxin (TTC), provided either as protein or encoded by non-viral naked DNA plasmid, binds pre-synaptic motor neuron terminals and can facilitate the retrograde axonal transport of desired therapeutic molecules to the CNS. Alleviated symptoms in animal models of neurological diseases upon delivery of therapeutic molecules offer a hopeful prospect for TTC therapy. This review focuses on what has been learned on TTC-mediated neuronal targeting, and discusses the recent discovery that, instead of being merely a carrier molecule, TTC itself may well harbor neuroprotective properties.

## 1. Introduction

### 1.1. Overview

Neurodegenerative disorders are a group of pathological conditions that typically originate from progressive dysfunction and loss of neurons or synaptic contacts in defined areas of the nervous system. For example, Alzheimer’s, Parkinson’s and Huntington’s diseases affect distinct parts of the central nervous system (CNS), whereas motor neuron diseases such as Amyotrophic lateral sclerosis (ALS) and spinal muscular atrophy (SMA) initiate peripherally by a loss of motor neural connection with the muscle. Although major steps in understanding the pathology and molecular mechanisms underlining these disorders have been taken, effective treatment has only been described in few. Besides the obvious task of finding the appropriate molecules or interventions with beneficial effect, one of the greatest challenges in development of treatments for neurodegenerative conditions relies on the fact that systemic delivery of potential therapeutics, such as growth factors or neurotrophins, may be ineffective due to short half-life and low bioavailability at the target tissue, as well as having potentially hazardous side effects [1,2]. The existence of blood-brain-barrier (BBB) normally precludes direct vascular delivery of large molecules to the CNS. It is, therefore, worthwhile to look for delivery methods that are minimally invasive and can be administered to the peripheral tissues but which may then specifically bear the therapeutic effect via distal neurons into the CNS. Traditionally these methods have relied on the ability of neurotropic viruses to serve as carriers, because they are taken up by the nerve termini and transported to the soma. However, although these may be resolved in the future, viral gene therapy may still involve complications, such as cargo molecule size restriction, as well as potential hazards related to genomic integration, that need to be considered [3]. 

Non-toxic tetanus toxin C-fragment (TTC) has been exploited as a molecular tool to specifically target motor neurons and has greatly facilitated studies of neuronal processes such as endocytosis, synaptic sorting and retrograde transport *in vitro* and *in vivo* [4]. Molecules may be fused with TTC without apparent loss of biological activity, which can provide valuable therapeutic tools for fighting neurodegeneration. Furthermore, recent evidence also suggests that TTC may harbor therapeutic properties itself [5]. This review focuses on what has been learned about TTC based therapies with special emphasis on motor neuron diseases. Besides the previously mentioned neurodegenerative conditions, the design of enzyme-replacement therapies for lysosomal storage disorders with neurological involvement may benefit from the trans-synaptic properties of TTC.

### 1.2. Tetanus Toxin

Tetanus neurotoxin (TeNT, also known as TeTx or tetanospasmin) is a protein produced by *Clostridium tetani*, an anaerobic bacterium whose spores are commonly found in soil and animal waste. TeNT is a causative agent of tetanus, a potentially fatal condition that affects the nervous system and is characterized by painful, uncontrolled muscle contractions [6]. It is synthesized as a single polypeptide and is post-translationally modified to produce light (L) and heavy (H) chains linked by disulfide bonds [7]. Although the binding and internalization of the toxin has not been systematically studied in different populations of neurons, TeNT shows remarkable affinity and specificity to neuronal terminals. It is internalized primarily by the motor neurons at the neuromuscular junction (NMJ) where it enters the axonal retrograde transport pathway and is subsequently transported to the neuronal soma in the CNS [4,8]. The L chain harbors a neurotoxic zinc metalloprotease activity which targets integral membrane protein VAMP/synaptobrevin, a synaptic vesicle-associated membrane protein required for neurotransmitter release [9,10]. Proteolytic cleavage of VAMP/synaptobrevin in the synapses of inhibitory interneurons (Renshaw cells) by the TeNT L chain, prevents the release of the neurotransmitters γ-aminobutyric acid (GABA) and glycine into the synaptic cleft, and results in sustained motor neuron excitation causing tetanic spasms. The H chain consists of two non-toxic fragments, H_N_ and H_C_. The H_N_ is thought to facilitate the translocation of the toxic L chain from endosomal compartment to the cytosol or otherwise modulate its trafficking route [11]. The 50 kDa carboxy-terminal H_C_ (hereafter called TTC) is required for neuronal cell binding and retrograde transport [12,13,14,15]. 

Neuronal transport [16], which normally carries enzymes and cellular organelles from distal axon terminals to the neuronal cell bodies, can be exploited by the TeNT to penetrate the BBB and to enter the CNS [8]. TeNT toxicity relies on the presence of L chain, but its targeting to the neuronal synapses is dependent solely on non-toxic TTC [15]. The internalization of both holotoxin and TTC initiates by interaction of TTC with neural gangliosides and specific (mainly uncharacterized) proteins on axolemmal infoldings associated with lipid microdomains of neuronal surface [17,18,19,20,21,22]. The binding is followed by clathrin-dependent endocytosis [23,24] and intracellular trafficking inside neutral endocytic vesicles [25]. The vesicles move retroaxonally towards the motor neuron cell body mainly using fast axonal transport that relies on both actin microfilaments and microtubule-interacting motor proteins [4,26,27]. From the soma, TTC may further reach the dendrites and can subsequently be transported to interneurons that synaptically connect with motor neurons [13,28].

Early studies of transsynaptic passage of radioactively labeled TeNT *in vivo* were limited by development of clinical tetanus and rapid death of the experimental animals [29,30]. Separation of toxicity and targeting functions by enzymatic cleavage or molecular cloning methods has allowed *in vivo* investigation of TTC internalization by neurons and their retrograde transport from the periphery to the central nervous system. Indeed, TTC has become an important tool to investigate neuronal physiology in a normal context and in conditions where neuronal function is affected by a disorder. 

## 2. TTC as a Neuronal Retrograde Tracer and Carrier of Therapeutic Molecules

Several molecules, including reporter genes and potential therapeutic molecules, have been successfully transported into neurons by coupling them with TTC (Table 1). The use of TTC as a carrier for therapeutic molecules to the CNS was first proposed by Bizzini and coworkers [12,17]. Since then, even large 150 kDa proteins have been shown to be efficiently internalized by TTC-mediated endocytosis. Here, we will first describe the various marker genes used to study TTC targeting function, neuroanatomy and physiology, and then proceed to the therapeutic approaches achieved by chimeric TTC constructs (protein or gene therapy) *in vitro* and in animal models of neurodegenerative diseases. 

### 2.1. TTC as a Tracer for Neuroanatomy and Cellular Physiology

Natural capability of TTC to employ neuronal retrograde transport machinery and transcytose to connecting higher order neurons has been exploited to investigate neuroanatomy and to trace physiological and molecular mechanisms of retrograde axonal trafficking. The applied methods include chemical or genetic conjugation of TTC to a histochemically of fluorescently detectable reporter molecules, as well as direct labelling of TTC with fluorochromes. Fluorochrome-labelled or chemically conjugated TTC-tracers may be delivered as protein injection. On the other hand, genetic fusion proteins have been administered either as a recombinant protein or as non-viral “naked DNA” plasmid [31]. In the latter method, the TTC hybrid-encoding plasmid is directly injected into the tissue where it is taken up by cells, transcribed as mRNA, translated to protein and secreted. Various routes of administration have been used *in vivo*, the most common being intramuscular injection.

More than 20 years ago it was shown that intramuscular administration of recombinant TTC conjugated with horse radish peroxidase (HRP-TTC) [13] could result in internalization of HRP activity by motor neurons and its axonal transport within cytoplasmic vesicles. Furthermore, TTC-conjugation was shown to radically improve neuronal internalization of even large proteins, such as human IgG [32] or glucose oxidase [33], and to enable neuronal delivery of DNA coupled to the TTC with polylysine bridge [34]. Similar findings were found later using TTC coupled with beta-galactosidase (β-gal-TTC) delivered intramuscularly either as β-gal-TTC fusion protein [35,36,37] or as direct “naked DNA” plasmid injection in *Xenopus* [38] and in mice [37]. It was also demonstrated that β-gal-TTC is transneuronally transported to second and higher-order interconnected neurons [36] and that the transport to motor neurons was dependent on neuronal activity [39]. Block of neuronal action potentials by surgical denervation or tetrodotoxin, inhibited the transmission of the tracer in the NMJ, whereas blocking acetylcholine stimulation of the post-synaptic muscle by botulinum toxin, which leaves nerve function intact, did not affect the transmission. 

To facilitate direct microscopic visualization, fusion protein of TTC and green fluorescent protein (TTC-GFP) has been successfully delivered to the neurons by using adenoviral vectors or intracerebral crafting of transfected neural cells [40], by transgenic methods, in mice expressing GFP-TTC hybrid gene under general and cell-type-specific promoters [41,42] as well as by direct intramuscular injection fusion protein [24,43,44]. Because the intensity of the signal is effectively diluted when TTC tracer proceeds towards CNS, the detection of fluorescently labeled tracer becomes progressively more challenged. For example, Perreault and coworkers [43] used intramuscular injection of TTC-GPF to trace central connections from a single hind-limb muscle in neonatal mice. Although spinal motor neurons were effectively labeled, the labeling of more central pre-motor neurons was restricted to the synaptic terminals and lacked signal in the cell soma. In another study, however, GFP-TTC fusion protein was readily detected in the soma of inhibitory interneurons [44] and the reason for this discrepancy remains unclear. Until the problems related to the weak signal have been resolved, the fine details of the higher order neuroanatomical connections are likely to rely on transgenic animals where high and more precise expression may be achieved, and even in this case anti-GFP antibody staining instead of direct fluorescent detection is usually required [41,42]. In the transgenic mice used by Makos and coworkers [41], the GFP-TTC tracer was coupled with an internal ribosome entry site driving the expression of β-gal, which provides an additional advantage as the cells of origin (β-gal positive) may be easily identified from those where GFP-signal has been acquired by transneuronal transport (β-gal negative). So far, GFP-TTC (or TTC-GFP) transgenics have been used successfully to trace neuronal connections in hippocampus [41], circuits that control locomotion and posture [44] and those that regulate sleep/wakefulness state [42].

**Table 1:** List of proteins conjugated to TTC, their conjugation method and models that have been used to study neuronal internalization *in vivo* and *in vitro*.

Construct	Fused Protein	Method ^1^	Model (Administration); *in vitro* Model ^2^	Reported Location/Transport, (effect)	References ^3^
TTC-HRP	Horse radish peroxidase	CC, RPb	Mouse (IM, IP, IC)	Coated vesicles, endosomes	[13,58]
TTC-HRP-hIgG	Horse radish peroxidase-hIgG	CC	Mouse (IM, IP)	MNs, CNS	[32]
GO-TTC	Glucose oxidase	CC	Mouse (IM)	MN terminals, CNS	[33]
β-Gal-TTC	β -galactosidase	RPb, ND	Mouse (IM), rat (IM), Xenopus (IM)	NMJs, MNs, CNS, 2nd and higher order neurons	[35,36,37,38,39]
GFP-TTC	Green fluorescent protein	RP, ND, TG	Mouse (IM, IC); mouse and rat primary culture neurons, rat spinal cord MNs	MN terminals, coated vesicles, endosomes, 2nd and higher order neurons	[24,35,40,41,42,43,44,68,99]
TTC-HEXA	β -N-acetylhexosaminidase-A	CC	Rat primary culture neurons, feline GM2 gangliosidosis neuronal culture	Endosomes, (enhances GM2 degradation - secondary lysosomes?)	[48]
SOD1-TTC	Cu/Zn superoxide dismutase	RPb	Mouse (IM, IC); murine neuroblastoma hybrid cell line	Intra-cytoplasmic vesicles, MNs, CNS, (no protection from oxidative injury)	[52,53,59,63]
SMN1-TTC	Survival motor neuron 1	RPb	Rat primary culture neurons	Neuronal surface, not internalized	[63]
CT1-TTC	Cardiotrophin-1	RPb	Mouse and rat primary culture neurons, rat spinal cord MNs	Intra-cytoplasmic vesicles, synaptic contacts, (induces CT1-dependent cellular processes)	[68]
Bcl-xL-GFP-TTC	B-cell lymphoma-extra large	RPb	Rat spinal cord MNs, dorsal root ganglion neurons	Internalized and transported to soma, (protects from apoptosis)	[72]
IGF-1-TTC	Insulin-like growth factor 1	RPb, RPi	Mouse, SOD1G93A mouse (IM, IT); mouse spinal cord MNs	Intra-cytoplasmic vesicles, MNs, CNS, (prevents muscle force decline with age, fails to improve SOD1G93A survival)	[79,80,81]
GDNF-TTC	Glial derived neurotrophic factor	CC, RPb, RPi	Mouse (IM, IT), SOD1G93A mouse (IM); mouse neuronal cell lines, rat axotomized MNs	MNs, CNS, (protect axotomized MNs, prolongs SOD1G93A survival)	[95,96,97]
BDNF-TTC	Brain derived neurotrophic factor	RPb	Mouse cortical neurons and neuroblastoma cell line	Internalized, (induces Akt pathway, inhibits apoptosis)	[98]

^1^ CC= chemical cross-link, RPb= bacterial recombinant protein, RPi= insect recombinant protein, ND= naked DNA plasmid, TG= transgenic animal. ^2^ IM= intramuscular injection, IP= intraperitoneal injection, IT= intrathecal injection, IC= intracerebral injection, MNs= motor neurons. ^3^ Please note that the references are given in the order that they appear in the text.

As for neuroanatomy, TTC has served as an important tool to investigate mechanistic aspects of neuronal traffic. Using paramagnetic iron beads coupled with TTC, it was shown [26] that small GTPase Rab7 plays an essential role as a functional marker of axonal retrograde carriers which transport neurotrophins and their receptors, such as brain-derived neurotrophic factor (BDNF), its receptor TrkB and the p75 neurotrophin receptor (p75NTR). These factors regulate synaptic strength and plasticity in mammalian nervous system as well as promote growth, maintenance and survival of their target neurons. This observation is interesting because it implicates that TTC exploits machinery that naturally occurs in nerve cells, and, therefore, may provide a more reliable delivery method for therapies aiming to increase trophic support for the neurons in neuronal diseases. Additionally, when coupled with visible markers such as fluorochromes, TTC may provide a useful tool for studies of axonal transport mechanisms in development and disease. This is of particular interest for those studying motor neuron diseases, since mutations perturbing retrograde transport have been shown to mimic hallmarks of human pathology [27,45]. Recently it has been demonstrated [46] that TTC can be fluorescently labeled without loss of its biological potency or immunoreactivity. TTC labeled with fluorochromes could be detected with whole-body molecular imaging technology allowing determination of *in vivo* retrograde transport to the CNS after intramuscular injection, as well as *ex vivo* histological mapping of the fate and transport rate of the tracer. Because of the non-toxicity and non-invasive administration, tracers of this class may become important agents for neurography in humans. For example, they may ease neuronal detection in complicated surgical procedures, aid visualization of neuronal circuits after injury, or function as a marker to evaluate therapeutic effects of neuroprotective of neurotrophic treatments.

Less is known about the mechanism of how TTC and its fusion variants are transported from the motor neuron dendrites to the more central interconnected axons. The existing data suggests that the internalization is mechanistically different in the presynaptic terminals of the NMJs and in those of centrally located axonal projections, such as inhibitory interneurons [10]. For example, in cultured hippocampal neurons TeNT is internalized by synaptic vesicle recycling, *i.e.*, in a depolarization- and calcium-dependent manner [47]; whereas in motor nerve endings the internalization is clathrin-dependent [23] and is not significantly altered by depolarizing conditions and is little affected by calcium [24]. The question also remains if intracellular trafficking of TTC molecules in the central interconnected neurons relies on similar retrograde transport apparatus as in the primary motor neurons [4,23,25,26]. These potential differences require further elucidation and are likely to be clarified when more sensitive methods for centrally interconnected neurons are developed. It is also worth mentioning that TTC may not work well as a tracer in neonatal animals where NMJs are not fully mature [43]. However, the potential of TTC to mediate retrograde transport to the CNS in adult animals is clearly established and has served as a starting point for studies investigating TTC-dependent trafficking of potentially therapeutic enzymes to the CNS.

### 2.2. TTC-mediated Neuronal Targeting of Metabolic Enzymes

Lost or altered metabolic enzyme activity underlies many neurodegenerative and neuromuscular diseases, including classical MNDs, some forms of peripheral neuropathy and neuronal lysosome storage diseases. When a single gene product is affected negatively by the condition, enzyme replacement therapy may offer a promising approach to compensate insufficient biological activity.

Tay Sachs disease (TSD) is an autosomal recessive human GM2 gangliosidosis characterized by lipid accumulation in the lysosomes of the nervous tissue and progressive deterioration of the brain and the spinal cord. TSD is caused by inactivating mutations in the gene for lysosomal GM2 ganglioside-degrading enzyme beta-hexosaminidase (HEXA) and represents an attractive target for enzyme replacement therapy. Human placental HEXA conjugated with TTC (but not HEXA alone) was found to be efficiently internalized by rat brain cells *in vitro* [48]. Furthermore, HEXA uptake was not enhanced by the presence of free TTC indicating the dependence on covalent linkage between the therapeutic molecule and the TTC. In cerebral cortex cell cultures from cat model of TSD, TTC-HEXA conjugates were able to reduce the accumulation of lysosomal lipids indicating that TTC-conjugation did not interfere with the enzymatic activity of HEXA. This also suggests that a significant part of the TTC-HEXA reached secondary lysosomes where GM2 gangliosides preferentially accumulate in these cells. While these results are promising with respect to non-viral treatment of lysosomal storage diseases with neurological involvement, they still await verification as a therapeutic tool in described animal models [49].

Cu/Zn superoxide dismutase (SOD1) is a cytosolic enzyme that converts superoxide radicals to molecular oxygen and hydrogen peroxide and thus protects cells from oxidative stress. SOD1 is mutated in 20% of the familial ALS patients [50] and increased SOD1 activity is neuroprotective in a mouse model of chemically induced Parkinson’s disease [51]. Recombinant fusion proteins of human SOD1 and TTC (SOD1-TTC), but not SOD1 alone, were shown to be efficiently internalized by the motor neurons and transsynaptically transported after intramuscular injections in mice [52]. Post injection, the molar excess of SOD1-TTC to the endogenous mouse SOD1 was estimated to be six-fold in motor neurons indicating efficient internalization. However, the activity of SOD1 in the fusion constructs was negatively affected, and it is not known to which subcellular location SOD1-TTC transported (endosomal or cytosolic). *In vitro*, SOD1-TTC molecules are ineffective in protection of cells from starvation-induced oxidative stress, possibly due to their location in the vesicles [53]. Although SOD1-TTC remains a potentially attractive neurotropic antioxidant, its beneficial *in vivo* effects have not yet been reported. Because SOD1 mutants associated with ALS are not hypomorphic but instead gain-of-function, wild-type SOD1 expression in ALS may not be beneficial at least in familial ALS [54,55]. However, SOD1 expression has been shown to be beneficial for recovery from spinal cord injury and ischemia in rats [56,57] and, therefore, may harbor alternative therapeutic use in post-traumatic treatment.

Although TeNT entry to the CNS is thought to be primarily mediated by binding to the motor neuron terminals at the NMJ, all neurons seem to be capable of binding both TeNT and TTC. Recombinant SOD1-TTC fusion protein used for a previous study [52] was also the first one used to evaluate the distribution and uptake of TTC-coupled protein after direct intracerebral injection [58] and cerebrospinal fluid infusion [59]. Although the metabolic effects of the SOD1 were not evaluated in the first study, TTC-SOD1 fusion protein showed vastly superior retention within brain tissue than SOD1 alone (or bovine serum albumin), and were distributed in synaptic and endosomal pattern suggesting retrograde transport [58]. In the latter study, it was demonstrated that TTC improved the stability of the SOD1, consistent with internalization by neurons. The delivered fusion protein was structurally intact and retained its SOD1 activity [59]. This suggests that, besides well-documented intramuscular administration, TTC may be suitable for intracerebroventricular or direct brain administration when less invasive methods are considered inefficient. This could be the case where the target neurons do not have projections outside the BBB or where specific centrally located parts of the brain are affected. However, the possibility that SOD1-TTC treatment is ineffective exists if, like *in vitro*, it is not released from the endosomal containment [53].

Spinal muscular atrophy (SMA), the most common genetic cause of infant mortality [60], is a recessive paralytic disease that results in selective loss of lower motor neurons. It is caused by mutations that reduce the amount of survival of motor neuron 1 (SMN1) [61], a protein that orchestrates the assembly of multiple protein-RNA complexes and contributes directly to mRNA splicing [62]. Increased levels of neuronal SMN1 may be beneficial for SMA patients, leading Francis and coworkers [63] to study the binding and internalization of genetic fusion of SMN1 and TTC (SMN1-TTC) in neuronal cell cultures. Because it is not clear if the passenger proteins may escape from the vesicular compartment after internalization by neurons, the authors included modified piece of diphtheria toxin to the SMN1-TTC molecule which they predicted to facilitate the export from the endosomal compartment to the cytosol. Unfortunately, collective evidence from these studies suggested that although all domains of the fusion protein retained their activity the internalization was prevented by the SMN1 domain. Importantly, this study implicated that the properties of the passenger molecule may negatively affect the internalization of the fusion protein.

### 2.3. TTC-mediated Delivery of Survival Factors, Growth Factors and Neurotrophins

Neuronal survival is dependent on constant exposure to neurotrophins which also mediate differentiation, growth, and apoptosis of neurons by binding to two types of cell surface receptors, the Trk tyrosine kinases and the p75NTR [45,64]. As mentioned earlier, TTC is transported inside the motor neurons in compartments including neurotrophins and their receptors [26], and may therefore serve as a “natural” signal to potentiate targeting of factors expected to enhance neuronal viability by inducing survival signals and by counteracting apoptotic cell death. 

Cardiotrophin-1 (CT1) is a cytokine of IL6 family that can increase motor neuron survival *in vitro* and *in vivo*. CT1 improves motor function, increases survival and protects NMJs in progressive motor neuropathy (pmn) mice, after adenoviral or naked DNA mediated delivery [65,66]. Additionally, intramuscular administration of adenoviral vector-encoded CT1 has been shown to be beneficial in the mouse model of SMA [67]. However, serious pleiotrophic side effects resulting from the high-level systemic or muscle administration, such as weight loss and cardiac hypertrophy, would preclude the use of CT1 for molecular therapy in human patients. To circumvent this problem, more targeted delivery of this cytokine to the nervous system was studied [68]. Genetic fusion of CT1-TTC (which also included GFP for direct visualization) efficiently bound and was internalized rapidly by cerebral neurons and spinal cord motor neurons *in vitro*, whereas the glia, hepatocytes or cardiomyocytes did not bind or uptake the recombinant protein. CT1-TTC promoted motor neuron survival in a dose-dependent manner *in vitro* and induced typical CT1 responses, including IL-6 secretion and transcriptional activation of its known target genes. This implies that TTC does not interfere with the endogenous function of the cytokine and that CT1-TTC could serve as a candidate for *in vivo* studies. TTC-conjugation may be particularly relevant to CT-1 since, unlike some other neurotrophic factors, it is not transported to neuronal dendrites and may be subjected to rapid lysosomal degradation [69], both of which could be positively modulated by TTC domain. 

Apoptosis plays a key role in death of neurons in neurodegenerative diseases [70]. Findings from patients and animal models of such diseases indicate strong contribution by altered mitochondrial metabolism, and respiratory chain-deficient cells are more likely to undergo apoptosis. Bcl-xL is an antiapoptotic protein whose expression is decreased in neurodegenerative conditions such as ALS [71]. Bcl-xL-TTC fusion protein (which also contained GFP for detection) was found to be specifically taken up by rat spinal cord motor neurons and dorsal root ganglion neurons and was transported to the cell bodies *in vitro* [72]. Importantly, fusion protein application improved cell survival and decreased apoptosis in response to glutamate-mediated toxicity. In the same study, it was reported that the positioning on the TTC to the carboxy-terminal side of the Bcl-xL-TTC fusion protein was an absolute requirement for internalization. Although Bcl-xl-TTC remains a promising candidate for exploration in animal models of neurodegenerative disease, studies investigating its effect *in vivo* are still pending.

Several neurotrophic factors and growth factors have been suggested for the treatment of motor neuron diseases. In ALS patients, however, the repeated subcutaneous injection of these factors as recombinant proteins may be complicated by their systemic toxicity due to their tropism to a variety of tissues and a poor bioavailability at the motor neuron terminals [73,74,75]. The latter may be affected by inefficient binding/internalization or short half-life of these factors. 

Insulin-like growth factor 1 (IGF-1) promotes growth of both skeletal muscles and motor neurons, and muscle-specific expression of IGF-1 can provide trophic support to the motor neurons. IGF-1 has been shown to decrease disease pathology and progression in animal models of ALS [76] and spinal and bulbar muscular atrophy [77], as well as alleviate symptoms in a mouse model of diabetic peripheral neuropathy [78]. Fusion protein consisting of IGF1 and TTC domain (IGF1-TTC) retain biological activity of both domains as demonstrated by increased mitogenic activity in L6 muscle cells as well as by *in vitro* and *in vivo* neuronal retrograde transport [79]. Furthermore, intramuscular administration of IGF1-TTC prevented specific force decline in aging mouse muscles, as well as attenuated age-related denervation of fast muscle NMJs [79,80]. However, although IGF-1 delivery to the spinal cord was massively improved by TTC domain, and it retained significant level of IGF-1 activity as measured by Akt phosphorylation, no positive effect could be demonstrated on the course of the disease in a mouse model of ALS either by intrathecal of intramuscular delivery [81]. This is consistent with previous demonstration that muscle-specific or CNS over expression of IGF-1 is not beneficial in ALS mice [82], suggesting that central or retrograde delivery of IGF-1 does not improve ALS symptoms. The efficacy of IGF1-TTC in other forms of disease where IGF-1 is expected to be beneficial is currently unknown.

Glial cell-derived neurotrophic factor (GDNF) potently promotes the survival, proliferation and differentiation of various types of neurons, including motor neurons [83,84,85,86,87]. Because penetration of GDNF into brain tissue from either the blood or the cerebro-spinal fluid is limited, direct delivery to the brain has been widely used despite being a highly invasive procedure. In rodent and primate models of Parkinson’s disease, GDNF is neuroprotective [88,89] but a number of clinical trials in which GDNF has been directly delivered to the brain of Parkinson’s disease patients have produced mixed, inconclusive results [90,91,92]. In a mouse model of ALS that expresses human SOD1 mutant (G93A), skeletal muscle-specific expression of GNDF increased survival of the spinal motor neurons, delayed the onset and progression of the disease, as well as increased life span [93]. However, expression in the CNS (astrocytes) carried out in the same study failed to demonstrate any protective effects, suggesting that retrograde delivery to the neurons was required for the therapeutic effects. In a rat model of ALS (SOD1-G93A), human neural stem cells genetically modified to release GDNF and surgically transplanted into CNS protected motor neurons but not their projections to the muscle [94]. In mice, the delivery of GDNF to the spinal motor neurons and spinal cord is improved by conjugation with TTC after intramuscular of intrathecal administration and, in both cases, the biological activity of GDNF is maintained [95,96]. GDNF-TTC was also shown to increase survival after experimentally induced damage rat spinal cord motor neurons [95]. Importantly, Ciriza and coworkers [97] demonstrated that genetically fused GDNF-TTC is antiapoptotic and induces Akt kinase survival pathway in cultured neurons, as well as improves activity and prolongs survival of the ALS (SOD1-G93A) mice. Although the effect of GDNF or TTC alone on lifespan was not demonstrated, these data suggest that intramuscular injection of GDNF-TTC may be beneficial at least in “dying-back” axonopathies such as ALS.

Brain derived neurotrophic factor (BDNF) is a member of the neurotrophin family of growth factors related to the canonical nerve growth factor, NGF. Genetic fusion of BDNF and TTC (BDNF-TTC) can induce neuronal survival Akt kinase pathway in mouse cortical culture neurons and inhibit apoptosis in mouse neuroblastoma cells [98]. Although protection from apoptosis was virtually identical compared with BDNF alone (but higher than TTC alone), mildly reduced potency or BDNF-TTC to activate Akt pathway may indicate negative effect on BDNF function by TTC. An alternative hypothesis is that TTC harbors antiapoptotic properties, which are potentiated by BDNF fusion. In this case, enhanced function of BDNF-TTC compared with TTC alone may derive from the fact that BDNF is able to promote TTC-internalization to the neurons in mature mouse motor neuron terminals [99]. Indeed, recent studies suggest that TTC itself harbors neuroprotective properties which are discussed in detail below.

## 3. Neuroprotective Properties of TTC Alone

Signaling through phosphatidylinositol 3-kinase (PI-3K), followed by PI-3K-mediated activation of serine/threonine kinase Akt is the key regulator of neuronal survival [64,100,101]. Additionally, protection from injury and cellular toxicity seems to be controlled by MAP kinase/extracellular signal-regulated kinase (MAPK/ERK) pathway [64]. Neuroprotection by neurotrophic factors derives from their potential to induce these pathways through their tyrosine kinase (Trk) receptors.

Binding of TTC to rat brain synaptosome activates Trk-dependent signaling, which mimics the action of their natural neurotrophic ligands [102,103]. TTC-mediated stimulation of Trk receptor in cultured cortical neurons and cerebellar granule neurons from rat [104] leads to activation PI-3K/Akt survival pathway as evidenced by fast and dose-dependent phosphorylation of Akt and its downstream target glycogen synthase kinase 3β (GSK3β). Additionally, TTC activated MAPK/ERK pathway as evidenced by phosphorylation of MAPK/ERK itself, two of its upstream kinases and downstream targets ribosomal protein S6 kinase (Rsk/S6K) and cAMP response element protein (CREB). Specific inhibition of Trk or PI-3K inhibited activation of both pathways by TTC. Additionally, TTC protects cerebellar granule neurons from apoptotic death induced by low extracellular potassium [104], as evidenced by largely preserved mitochondrial function, decreased nuclear fragmentation and reduced activation of pro-caspase 3. Again, the effects on apoptosis were dependent on activated PI-3K/Akt and MAPK/ERK pathways. Although detailed mechanism on how TTC activates Trk receptor remains to be elucidated, it is likely to bear similarities to those of growth factors, and it is conceivable that Akt and MAPK/ERK activation by TTC enhances its endocytosis by the neurons. Phosphorylated MAPK/ERK, as well as neurotrophin receptor p75NTR, TrkB, NGF and BDNF, has also been shown to be present in TTC positive organelles during retroaxonal transport [26,105].

The evidence pointing towards antiapoptotic properties of TTC is not only limited to *in vitro* studies. Parkinson’s disease is caused by degeneration of nigrostriatal dopaminergic neurons which results in dopamine deficiency and clinical features involving slowness of movements, tremor and rigidity. Parkinsonism can be induced in experimental animals by 1-methy-4-phenyl-1,2,3,6-tetrahydropyridine (MPTP), whose metabolite MPP^+^ specifically destroys dopaminergic neurons. In cerebellar granule neurons *in vitro*, TTC protects against MPP^+^ by enhanced protection against apoptosis [106]. In a rat model of Parkinsonism, where striatal lesions have been chemically induced *in vivo* by MPP^+^, striatal administration of TTC before MPP^+^ infusion resulted in significant amelioration of motor deficits and restored the dopamine levels to those of controls [107]. These results are promising because they may offer an alternative to the otherwise effective neurotrophins such as GDNF, which have poor penetration through BBB. Given that early diagnosis of Parkinson’s disease will be facilitated in the future by novel diagnostic markers, TTC administration could serve as an early intervention while a significant number of dopaminergic neurons remain healthy.

Neuronal death after ischemia, a condition that temporarily deprives the brain of oxygen and glucose, has been shown to rely on similar mechanisms observed in ALS patients including glutamate mediated excitotoxicity and oxidative stress. Similarly, cardiac surgery may lead to severe oxidative stress due to the formation of oxidation products generated during ischemia and reperfusion [108]. In a model of cerebral ischemia in Mongolian gerbils, pre-treatment with intramuscular administration of TTC, by non-viral naked-DNA method, protected neurons by reducing ischemia-induced oxidative stress markers such as nitric oxide, superoxide, lipid peroxidation. Additionally, although TTC *per se* had no effect on motor response, it suppressed characteristic hyperlocomotion in the post-ischemic animal [109]. Therefore, TTC administration may serve as a promising candidate to treat post-ischemic neuronal damage or as a preventive treatment before ischemia-inducing operations such as cardiopulmonary bypass surgery.

## 4. Future Directions

The evidence above infers that TTC-based methods may serve as promising alternatives to viral gene delivery for neuronal gene therapy. However, before these therapies are applicable to humans, problems related to efficient delivery methods and potential immunogenicity of TTC need to be addressed. Additionally, there is a demand for optimization of the non-viral naked DNA method to increase expression levels which, at the moment, may not be sufficient for administration to large muscles (although this has been shown to be effective in mice). Finally, in future TTC-mediated neuronal gene therapy experiments, it is crucial to include careful controls to investigate the potential TTC-specific or synergistic effects with the passenger molecules.

Although clear benefits of TTC conjugation for efficient and specific neuronal targeting have been demonstrated, it is becoming clear that, the optimal organization of the fusion modules needs to be determined for each molecule. Genetic fusion of trophic factors or enzymes to the TTC may alter their conformation thus undermining the natural properties, for example, by blocking their active site by steric hindrance. Alternatively, the efficiency of the TTC to promote retrograde transport could be affected by the passenger molecule resulting in insufficient internalization or altered trafficking. For example, with different domain organizations of SOD1-TTC, the activity of the enzyme was affected but the internalization and transport in the neurons was not [52]. In contrast, the internalization of Bcl-xL-TTC depended on c-terminal location of the TTC domain [72]. Furthermore, the internalization of SMN1-TTC, but not its neuronal binding, seemed to be inhibited by the SMN1 itself, revealing that not all proteins may be suitable for TTC-mediated neuronal uptake [63]. Finally, co-administration of neurotrophic factors such as BDNF seems to enhance internalization of TTC by the motor neuron terminals [99]. Therefore, to ensure maximal efficiency, it would be advisable to address *in vitro* internalization and transport kinetics as well as activity of the passenger molecule before *in vivo* animal studies.

The question of how much the passenger protein may influence the intracellular location remains, because the internalized TTC is largely endosomal. TTC-containing retrograde organelles display a neutral pH [25] which protects their cargo from degradation and allows long-distance axonal transport. Because this compartment is shared by neurotrophins and their receptors [105], diseases where neurotrophic stimulation is likely to alleviate pathology would be expected to benefit from TTC-fusion in a most straightforward manner. In contrast, if naturally cytosolic passenger molecules are bound to the membranes or trapped within endosomal compartment they may be ineffective even if their activity *per se* is maintained. SOD1 is normally cytosolic, and its vesicular location (as is the case with SOD1-TTC) may prevent its efficient function in free radical defence [53]. The situation may be even more complicated when trans-membrane transport is required to target the passenger molecule to a specific subcellular location, such as to the nucleus, mitochondria or lysosomes. In this case, at least partial delivery of TTC-HEXA to the secondary lysosomes is suggested by its capability to reduce the accumulated lysosomal substrates in the cortical cell cultures of feline TSD model [48]; whereas neuroprotective effects of Bcl-xL-TTC [72] may indicate mitochondrial targeting. Although this evidence is indirect, it may suggest that natural subcellular location of the passenger molecule can influence the route of TTC by diverting it from its normal transneuronal transfer pathway. If the release of the passenger molecule from the endosomal compartment is regarded important it may be facilitated by a fusion with a membrane translocating domain, such as that of diphtheria toxin [63,109]. Therefore, detailed studies of factors that facilitate routes to specific subcellular compartments (endosomal, lysosomal, mitochondrial, cytoplasmic or nuclear) are warranted.

TTC is truly neurotropic *in vitro* and *in vivo*, when delivered as protein or naked-DNA using various administration routes. However, membrane binding and internalization of TeNT holotoxin is more efficient than that of TTC alone [111]. Design of modified TTC-based carrier proteins containing additional non-toxic molecular domains may enhance its neurotropic properties and, therefore, the ability to efficiently deliver therapeutic proteins to the neurons. On the other hand, translation of TTC-mediated therapy to the clinical practice may be hindered due to immunogenic reactions towards TTC [112]. Most individuals in the developed world are vaccinated against tetanus, and even for those that are not, treatments in humans are expected to be long term when natural immunogenicity of TTC may become a problem. However, uptake of TTC by nerve terminals from an intramuscular depot is rapid and is not blocked by immunization against tetanus toxoid in mice [113]. Rapid neural internalization may protect TTC and conjugated passenger molecules by making them inaccessible to the antibodies. In fact, it has been suggested that circulating antibodies may even facilitate the specificity of the TTC-administration by inactivating molecules that potentially escape neuronal intake [114]. Additionally, modified ganglioside-binding domains of TTC that lack immunodominant epitopes may serve as an alternative strategy to circumvent TTC immunogenicity [115] (see also Dobrenis *et al.*, 2009 – Abstract 38 at Lysosomal Disease Network World Symposium 2010) provided that these re-designed TTC-molecules retain their neurotropic and retrograde transport properties. 

Although rapid axonal transfer of TTC to the CNS, especially in the large-diameter fibers, has been demonstrated [46,116], for the treatment of neurological disorders in humans it may be more critical to address the methods of administration that result in sufficient and pertinent expression of the therapeutic molecules in all or most neurons affected by the disease. It is evident that when balancing the efficient production and safe, non-invasive administration on one hand, and sufficient expression level and duration on the other, both TTC-mediated and viral delivery methods in their current state require further development. 

Because TTC is able to stimulate signaling pathways that increase neuronal survival, the question remains of how much the reported effects in general can be ascribed to the passenger molecules and how much to the TTC-carrier itself. As proposed by Mendieta and coworkers [107], the antiapoptotic effect of TTC-Bcl-xL could be at least partially due to TTC carrier and not the cargo. The same applies with all studies where TTC alone was not used as a negative control, particularly those that demonstrate activation of neuronal survival pathways. It is also necessary to recognize the fact that not all studies that included “TTC alone” control report enhancement on neuronal survival signaling or TTC-dependent effects in disease models [79,80,81,95,96]. Therefore, it is feasible that TTC-dependent promotion of neuronal survival depends on variables such as cell types used for *in vitro* activation of signaling pathways, as well as route and dose of administration *in vivo*, which may differ between laboratories. In future studies, it is advisable to include TTC itself as a control to preclude its effect from those of putative therapeutic molecules.

Another, perhaps related question is: Does the stimulation of retrograde transport *per se* contribute to the potential therapeutic properties of TTC? Defects in retrograde transport may characterize many neurodegenerative diseases [45,117,118] and experimentally induced defects in this cellular process replicate key features of human neurodegenerative pathology [27,119]. Therefore, stimulation of retrograde machinery may be beneficial in some conditions. Recent advances in molecular level live tracking techniques [120] may facilitate studies of the retrograde transport-stimulating actions of TTC and TTC-conjugates.

Finally, TTC may serve to target non-proteinous molecules, such as DNA or drugs, specifically to neurons. TTC-conjugated nanoparticles made from biodegradable polymers can selectively target neuroblastoma cells as opposed to liver or endothelial cells *in vitro* [121]. Similarly, self-assembled ternary vectors consisting of poly (ethylene imine) and TTC protein have been used to target DNA plasmids to dorsal root ganglion neurons *in vitro*, where they can elicit the expression of plasmid-encoded gene [122]. If shown to function *in vivo*, these vectors have a substantial potential to serve as retrograde transport-exploiting drug/gene delivery vehicles that may reduce the potential hazards associated with systemic drug delivery.

## 5. Conclusions

TTC offers a promising tool for specific targeting of neurons. It can be used either as a neuroanatomical and functional tracer, or administered directly by protein injection or by non-viral naked-DNA methods, to carry therapeutic biological activity into the CNS. Growing evidence suggests that TTC may itself harbor neuroprotective properties, which opens attractive avenues for future research on its potential use to alleviate neurological disorders. Future modifications of TTC molecule, as well as development of TTC-based therapeutic interventions, are likely to further enhance its efficacy and applicability as a molecular neurotrophic courier.
